# Fuzzy clustering of 24–2 visual field patterns can detect glaucoma progression

**DOI:** 10.1371/journal.pone.0309011

**Published:** 2024-09-04

**Authors:** Hwayeong Kim, Sangwoo Moon, Joohwang Lee, EunAh Kim, Sang Wook Jin, Jung Lim Kim, Seung Uk Lee, Jinmi Kim, Seungtae Yoo, Jiwon Lee, Giltae Song, Jiwoong Lee

**Affiliations:** 1 Department of Ophthalmology, Pusan National University College of Medicine, Busan, Korea; 2 Department of Ophthalmology, Pusan National University Yangsan Hospital, Pusan National University School of Medicine, Yangsan, Korea; 3 Department of Ophthalmology, Haeundae Paik Hospital, Inje University College of Medicine, Busan, South Korea; 4 Department of Ophthalmology, Dong-A University College of Medicine, Busan, Korea; 5 Department of Ophthalmology, Busan Paik Hospital, Inje University College of Medicine, Busan, Korea; 6 Department of Ophthalmology, Kosin University College of Medicine, Busan, Korea; 7 Department of Biostatistics, Clinical Trial Center, Biomedical Research Institute, Pusan National University Hospital, Busan, Korea; 8 Division of Artificial Intelligence, Department of Information Convergence Engineering, Pusan National University, Busan, Korea; 9 Center for Artificial Intelligence Research, Pusan National University, Busan, Korea; 10 School of Computer Science and Engineering, Pusan National University, Busan, Korea; 11 Biomedical Research Institute, Pusan National University Hospital, Busan, Korea; Xidian University, CHINA

## Abstract

**Purpose:**

To represent 24–2 visual field (VF) losses of individual patients using a hybrid approach of archetypal analysis (AA) and fuzzy c-means (FCM) clustering.

**Methods:**

In this multicenter retrospective study, we classified characteristic patterns of 24–2 VF using AA and decomposed them with FCM clustering. We predicted the change in mean deviation (MD) through supervised machine learning from decomposition coefficient change. In addition, we compared the areas under the receiver operating characteristic curves (AUCs) of the decomposition coefficient slopes to detect VF progression using three criteria: MD slope, Visual Field Index slope, and pointwise linear regression analysis.

**Results:**

We identified 16 characteristic patterns (archetypes or ATs) of 24–2 VF from 132,938 VFs of 18,033 participants using AA. The hybrid approach using FCM revealed a lower mean squared error and greater correlation coefficient than the AA single approach for predicting MD change (all *P* ≤ 0.001). Three of 16 AUCs of the FCM decomposition coefficient slopes outperformed the AA decomposition coefficient slopes in detecting VF progression for all three criteria (AT5, superior altitudinal defect; AT10, double arcuate defect; AT13, total loss) (all *P* ≤ 0.028).

**Conclusion:**

A hybrid approach combining AA and FCM to analyze 24–2 VF can visualize VF tests in characteristic patterns and enhance detection of VF progression with lossless decomposition.

## Introduction

Visual field (VF) defects occur due to various causes, and are associated with decreased daily functioning and quality of life [1–3]. Glaucoma is the most common disease causing VF loss except for cataracts, followed by degenerative myopia, non-glaucomatous optic nerve damage, and age-related macular degeneration [1, 2]. In addition to ophthalmic conditions, diseases such as stroke or brain tumors can cause VF loss [4, 5]. To detect VF defects and monitor their progression, 24–2 standard automated perimetry has become the clinical standard [6, 7]. Different methods have been proposed to classify VF defects [8]; however, detecting VF loss and its progression remains challenging for clinicians.

A study using machine-learning classifiers to diagnose glaucoma using VF tests was first introduced in 1994 [9]. Since then, several studies have been conducted using various classifiers [10, 11]. Among those studies, one group analyzed the patterns of 24–2 VF tests using archetypal analysis (AA) [11]. Seventeen patterns of VF defects, called archetypes (ATs), were identified, and each patient’s VF test was decomposed into 17 ATs [11]. AA is an unsupervised artificial intelligence technique used to analyze characteristic patterns in a dataset [12]. AA discovers representative features of the data by estimating the principal convex hull of a dataset. However, representing all the data as a convex combination in AA decomposition in a high-dimensional space may result in projection loss [13].

To overcome the limitations of projection loss, we propose a hybrid unsupervised approach that combines AA and fuzzy c-means (FCM), one of the most popular fuzzy clustering algorithms. Owing to its easy implementation and simplicity, FCM has become an important tool for pattern recognition [14, 15]. Especially, FCM and its modified algorithms are the most frequently used technique in medical image segmentation such as magnetic resonance imaging for its accuracy [16–19]. However, few studies have applied the FCM in the field of ophthalmology [20, 21]. Previous studies employed the FCM clustering algorithm to classify features of the optic disc in retinal fundus images for glaucoma diagnosis, which reported superior diagnostic efficacy of the FCM algorithm [20, 21].

In our previous study (in preprint status: Yoo et al., Research square, August 08, 2022, doi:10.21203/rs.3.rs-1909859/v1), we applied the FCM algorithm to recognize characteristic patterns of 10–2 VF and it was the first attempt to analyze VF using FCM clustering algorithm. FCM decomposition demonstrated its superiority over AA decomposition through lossless decomposition for representing 10–2 VF. Thus, we can theorize that FCM decomposition can represent characteristic 24–2 VF pattern of individual patient more accurately than does AA.

We aimed to classify the characteristic 24–2 VF patterns and decompose the VF of individual patients applying the hybrid approach using both FCM and AA. Then we compared the performance of detecting VF progression between the FCM and AA decomposition method.

## Materials and methods

This retrospective study was conducted according to the principles of the Declaration of Helsinki. The VF data of patients that had or were suspected to have glaucoma were collected from Pusan National University Hospital, Kosin University Gospel Hospital, Dong-A University Hospital, Busan Paik Hospital, and Pusan National University Yangsan Hospital between 1, June 2004 and 31, January 2021. The study protocol was approved by the institutional review boards (IRBs) of Pusan National University Hospital (2203-018-113), Kosin University Gospel Hospital (2018-12-028), Dong-A University Hospital (22–074), Busan Paik Hospital (2023-11-179), and Pusan National University Yangsan Hospital (05-2018-172). The requirement for patient consent was waived by the IRB due to the retrospective nature of the study. The data was accessed for research purpose, and the access periods are as follows: Pusan National University Hospital (25, March, 2022 to 31, March, 2024), Kosin University Gospel Hospital (30, January, 2019 to 31, March, 2024), Dong-A University Hospital (25, April, 2022 to 31, March, 2024), Busan Paik Hospital (11, December, 2023 to 31, March, 2024), and Pusan National University Yangsan Hospital (16, October, 2018 to 31, March, 2024). The data was de-identified and authors did not have access to information that could identify individual participants.

Automated perimetry was performed using a Humphrey Visual Field Analyzer 750i instrument (Carl Zeiss Meditec, Dublin, California, USA) with the Swedish interactive thresholding algorithm standard 24–2. Considering the learning effect, the first two VF tests for each eye were excluded [22, 23]. The reliability criteria for VF selection were a fixation loss rate ≤ 33%, a false-negative rate ≤ 20%, and a false-positive rate ≤ 20% [24–26].

**Fig 1:**
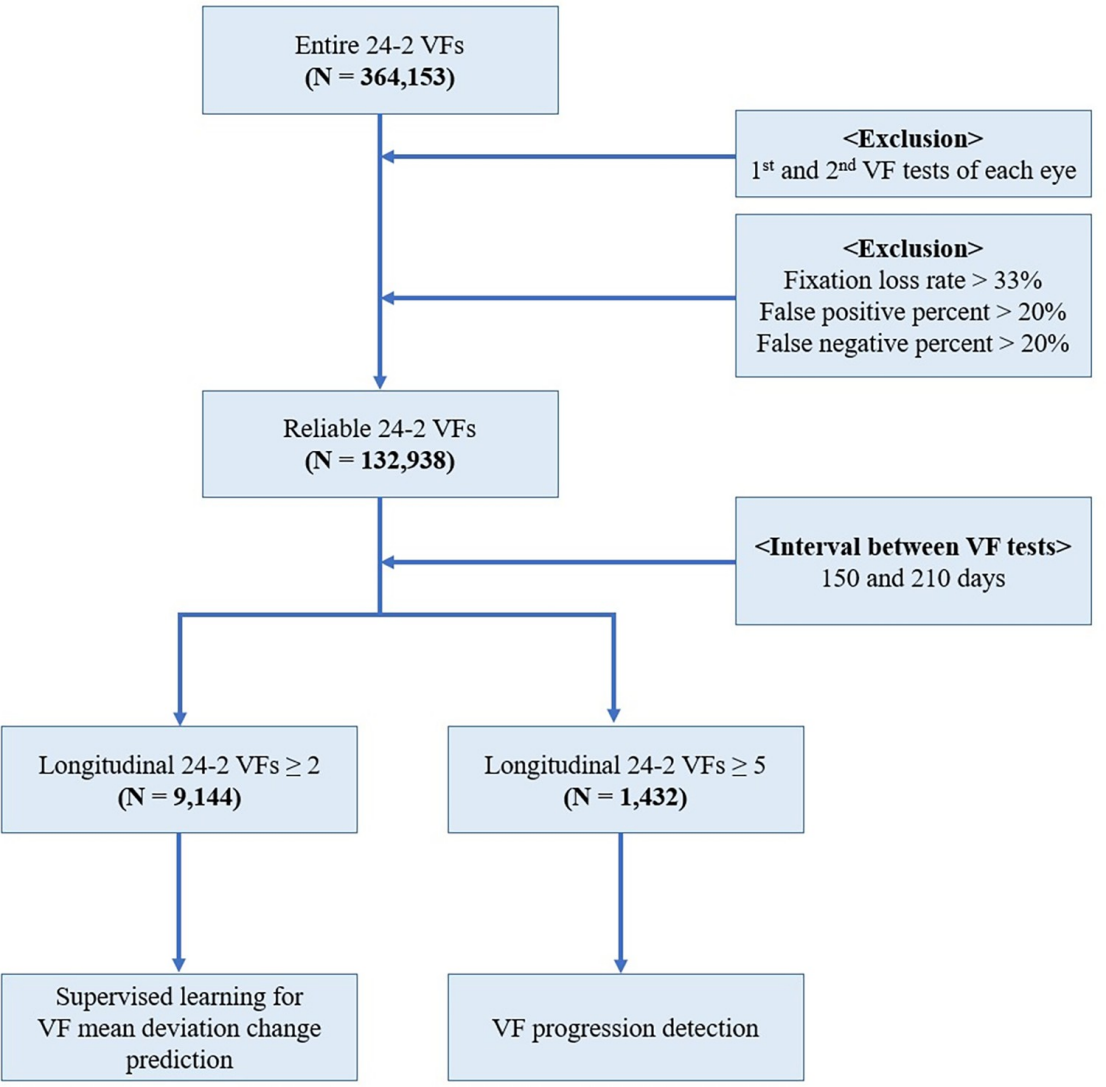
Flow chart of the analyzed data. The first two 24–2 visual field (VF) tests of each eye were excluded, considering the learning effect. Reliable VFs were included based on reliability criteria and were further divided into subgroups for longitudinal analyses: VF mean deviation (MD) change prediction (presented in Table 4) and glaucoma progression detection (presented in Table 5).

We obtained 132,938 reliable 24–2 VF tests from 364,153 tests. For longitudinal analyses, eyes with at least two reliable 24–2 VFs were selected to predict the mean deviation (MD) change from the decomposition coefficient change. In addition, eyes with at least five reliable 24–2 VFs and 3 years of follow-up were selected to compare the diagnostic ability of VF progression between the FCM and AA decomposition methods. The interval between each VF test used for the longitudinal analyses ranged from 150 to 210 days. If both eyes met the inclusion criteria, one eye was randomly selected for longitudinal analysis (Tables 1–3) (Fig 1).

**Table 1:** Demographic characteristics of the entire study samples.

Characteristics	
Total number of 24–2 visual field tests	132,938
Number of eyes (patients)	32,553 (18,033)
Age, mean ± SD (years)	55.5 ± 17.0
24–2 MD, mean ± SD (dB)	−7.4 ± 8.4
24–2 PSD, mean ± SD (dB)	4.8 ± 4.0
24–2 VFI, mean ± SD (%)	81.9 ± 26.1

MD = mean deviation; PSD = pattern standard deviation; SD = standard deviation; VFI = Visual Field Index

**Table 2:** Demographic characteristics of study sample selected for longitudinal analysis with two or more reliable 24–2 visual field tests.

Characteristics	
Total number of 24–2 visual field tests	9,144
Number of 24–2 visual field tests per eye, mean ± SD	2.7 ± 1.3
Number of eyes (patients)	3,449 (3,449)
Age, mean ± SD (years)	54.3 ± 16.8
Initial 24–2 MD, mean ± SD (dB)	−6.7 ± 7.8
Initial 24–2 PSD, mean ± SD (dB)	4.9 ± 4.1
Initial 24–2 VFI, mean ± SD (%)	83.6 ± 23.8

MD = mean deviation; PSD = pattern standard deviation; SD = standard deviation; VFI = Visual Field Index

**Table 3:** Demographic characteristics of study sample selected for longitudinal analysis with five or more reliable 24–2 visual field tests.

Characteristics	
Total number of 24–2 visual field test	1,432
Number of 24–2 visual field tests per eye, mean ± SD	6.3 ± 1.5
Number of eyes (patients)	229 (229)
Follow-up times, mean ± SD (months)	31.1 ± 9.2
Sex, Female (%)	113 (49.3%)
Age, mean ± SD (years)	54.7 ± 15.6
Initial 24–2 MD, mean ± SD (dB)	−5.4 ± 5.6
Initial 24–2 PSD, mean ± SD (dB)	5.0 ± 4.6
Initial 24–2 VFI, mean ± SD (%)	87.0 ± 17.7
MD slope, mean ± SD (dB/year)	−0.2 ± 0.9
VFI slope, mean ± SD (%/year)	−0.7 ± 2.8
Diagnosis	
Glaucoma suspect	19
Primary open angle glaucoma	180
Pseudoexfoliation glaucoma	6
Primary angle closure glaucoma	11
Secondary glaucoma	9
Others	4

MD = mean deviation; PSD = pattern standard deviation; SD = standard deviation: VFI = Visual Field Index

We first identified ATs representing patterns of the 24–2 VF tests using AA based on total deviation values (TDVs) [11]. We set the number of ATs considering reconstruction errors and clinical interpretation. In contrast to the previous studies, [11] we applied FCM to the decomposition process to prevent projection loss that AA inevitably has.

If we assume that the data set is {1,2,…*i*,…,n} and the clusters are {1,2,…,K}, then the weighted sum of the probabilities for data point *X*_*i*_ to belong to each cluster *C*_1_, *C*_2_,…,*C*_*K*_ is equal to 1.


$$\sum_{k=1}^{K}W_{ik}=1$$ (1)

A cluster C_*k*_ contains one or more data points, and the weighted values of data points within a cluster cannot all be 0 or all be 1 (If they were all 0 or all 1, the utility of cluster analysis would be lost). If there are *n* data points, the sum of weighted values for a single cluster must fall within the following range.


$$0<\sum_{i=1}^{n}W_{ik}<n$$ (2)

The symbol ‘||||’ denotes the Euclidean distance, where $|\left|p-q\right||=\sqrt{\left(p-q)*(p-q\right)}$. The weight associated with each cluster can be expressed by the Eq (3). *w*_*ij*_ represents the weight of the *i*-th data point in cluster *j*. The denominator signifies the distance between the *i*-th data point and all centroids.

$$w_{ij}=\frac{1}{\sum_{k=1}^{K}{\left(\frac{\left|\left|x_{i}-c_{k}\right|\right|}{\left|\left|x_{i}-c_{j}\right|\right|}\right)}^{\frac{2}{P-1}}}$$ (3)

$$where\;x_{i}=i-th\;data\;point\;c_{j}=j-th\;archetype$$

The centroid of each cluster can be obtained using the Eq (4) (For the *k*-th cluster).


$$c_{k}=\frac{\sum_{i=1}^{n}W_{ik}^{P}X_{i}}{\sum_{i=1}^{n}W_{ik}^{P}},\;k=1,2,\ldots ,K$$ (4)

In Eq (4), *W*_*ik*_ represents the weight for data point *i* in the *k*-th cluster and *X* represents the data vector. *P* is a hyperparameter controlling the degree of fuzziness in fuzzy clustering; as its value increases, the clustering becomes more blurred.

Ultimately, Fuzzy Clustering aims to minimize the Eq (5).


$$argmin\;SSE=\sum_{k=1}^{K}\sum_{i=1}^{n}w_{ik}^{P}{\left|\left|x_{i}-c_{j}\right|\right|}^{2}$$ (5)

We predicted the 24–2 VF MD changes using the FCM and AA decomposition coefficient differences for eyes with at least two reliable 24–2 VF tests, respectively. The differences in the FCM decomposition coefficients were calculated from the baseline coefficient, similar to MD change, which was calculated by subtracting baseline MD from MD at each visit. To predict MD change, we built machine learning models using K-nearest neighbor, random forest, and light gradient boosting machine. We trained the prediction models using 4,040 of the 5,772 instances as the training dataset. We evaluated the performance of the models using the remaining instances. We measured the mean squared error (MSE) and the Pearson correlation coefficient (PCC) formulated in Eq (6) as predictive evaluation metrics. The models were built 30 times using different partitions of the training and test datasets, and a paired t-test was performed to determine the statistical significance of our FCM decomposition for building the 24–2 VF MD change-prediction models relative to AA decomposition (note that the 24–2 VF MD change-prediction models were also trained using the AA decomposition for performance comparison) [27].

$$MSE=\frac{1}{N}\sum_{i=1}^{N}{\left(y_{i}-x_{i}\right)}^{2}\;and\;PCC=\frac{\sum_{i=1}^{N}(x_{i}-\bar{x})(y_{i}-\bar{y})}{\sqrt{\sum_{i=1}^{N}{\left(x_{i}-\bar{x}\right)}^{2}}\sqrt{\sum_{i=1}^{N}{\left(y_{i}-\bar{y}\right)}^{2}}}$$ (6)

$$where\;x_{i}=predicted\;MD\;change(by\;AA\;and\;FCM)\;and\;y_{i}=actual\;MD\;change$$

For eyes with at least five reliable 24–2 VFs, an ordinary least squares analysis of the decomposition coefficient over time was performed for each AT. The slope of the regression lines was defined as the rate of change of decomposition coefficient for each AT, as determined by AA or FCM, respectively. Three progression criteria were used to define VF progression: MD slope, Visual Field Index (VFI) slope, and pointwise linear regression (PLR). The slopes of MD and VFI over time were calculated using linear regression. If the slope was negative with a *P*-value < 0.05, the patient was classified as progressing [28–30]. PLR of the TDVs at 52 test locations over time was determined. If the regression slope for the TDV of three individual points was ≤ −1.0 dB/year with a *P*-value < 0.01, the VF was determined to be progressing [31].

Receiver operating characteristic (ROC) curves were generated by plotting sensitivity against 1-specificity, and the areas under the ROC curves (AUCs) were used to evaluate diagnostic performance for VF progression. The AUCs of the decomposition coefficient slopes against time of each AT obtained using the AA (slope_AA_) and FCM (slope_FCM_) were calculated for each progression criterion.

All statistical analyses were performed using R software (version 4.0.5; R Project for Statistical Computing, Vienna, Austria) and MedCalc (version 22.006; MedCalc Inc., Mariakerke, Belgium). *P*-values < 0.05 were considered statistically significant.

## Result

### 1. Representation of characteristic 24–2 VF loss patterns using 16 ATs

**Fig 2:**
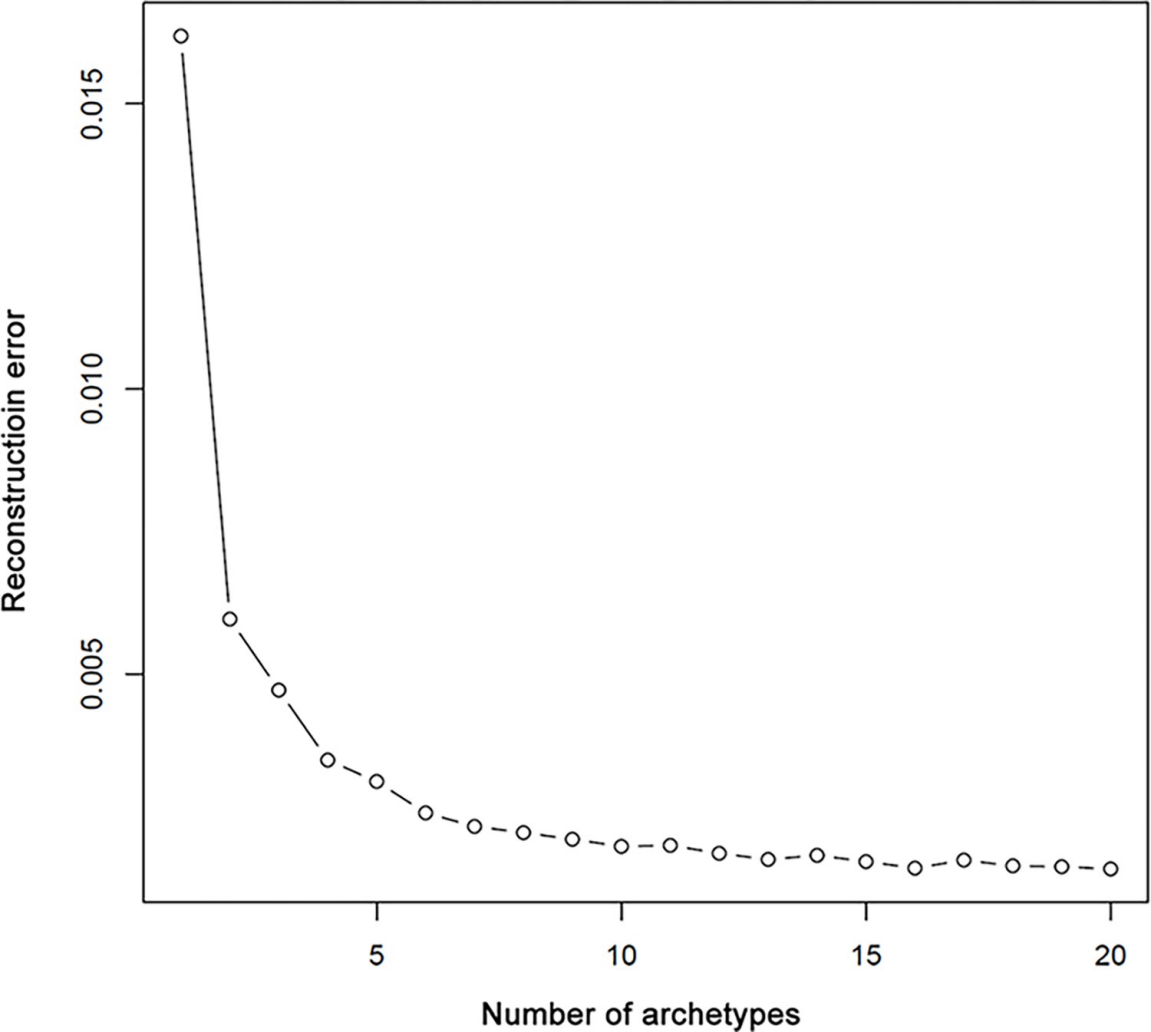
Scree plot in archetypal analysis. The number of archetypes (x-axis), the reconstruction error of the test set (y-axis). As the capacity to represent data increases, the reconstruction error naturally tends to decrease.

The number of ATs (denoted as *k*) was determined using AA. To determine the optimal *k*, we observed changes in the reconstruction errors in the test dataset while adjusting the number of ATs in the training dataset using five-fold cross-validation. Fig 2 illustrates the reconstruction errors according to the change in *k* values from 1 to 20. The reconstruction errors decrease as *k* increases; however, excessive ATs may make the clinical interpretation of VF patterns difficult. Therefore, we set the number of ATs to 16, which is clinically interpretable with a low reconstruction error.

**Fig 3:**
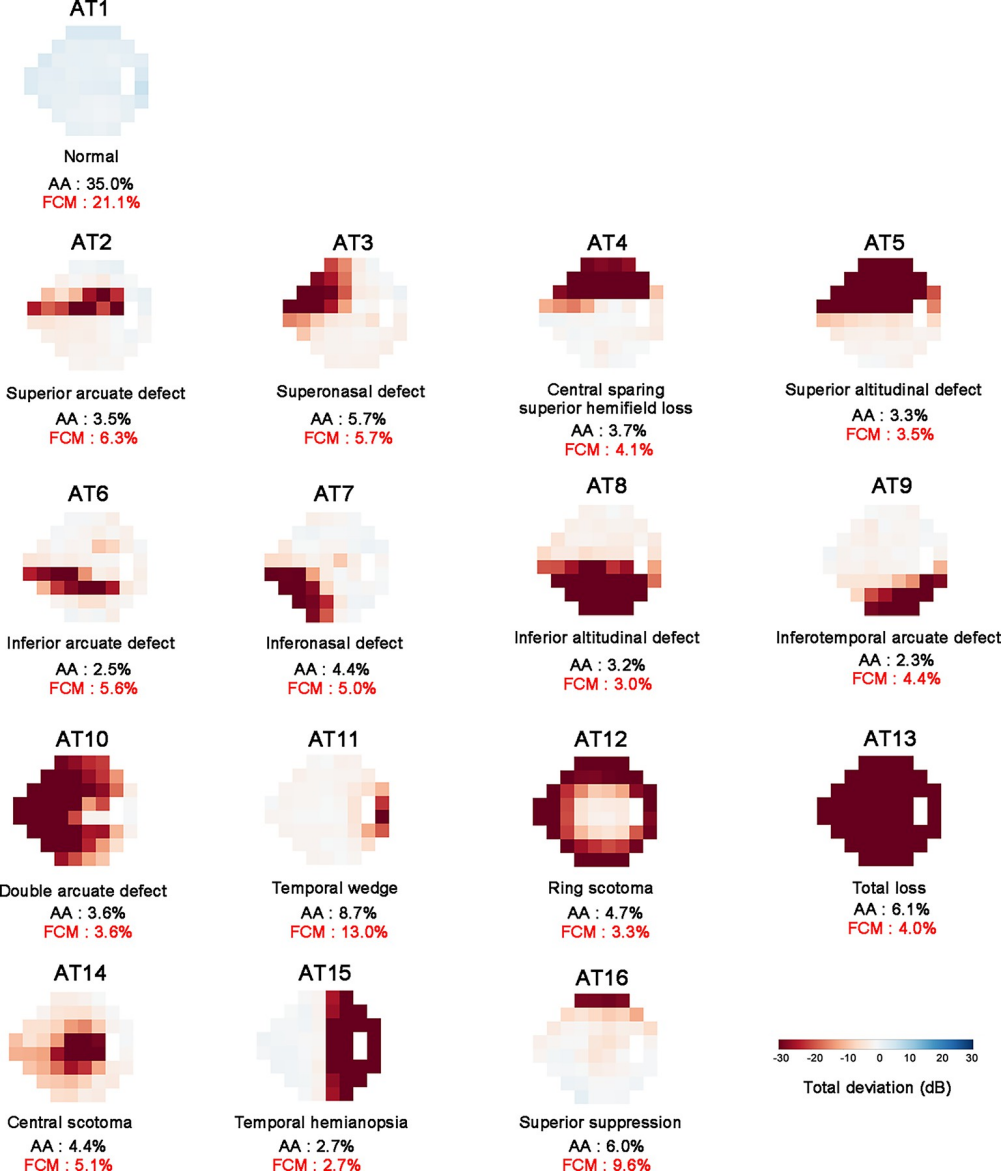
Sixteen representative 24–2 visual field patterns determined by archetypal analysis based on total deviation value (TDV). Red indicates low TDV (deterioration), and white or blue indicates high TDV (normal). Relative mean decomposition ratios for two methods are shown below each archetype for comparison. Note that all visual fields are plotted in right-eye format. AT = archetype; AA = archetypal analysis; FCM = fuzzy c-means.

AA was performed using 132,938 VF tests in 32,553 eyes of 18,033 patients. The mean ± standard deviation (SD) of age and 24–2 MD was 55.5 ± 17.0 years and −7.4 ± 8.4 dB, respectively (Table 1). Fig 3 illustrates 16 characteristic 24–2 VF patterns determined by AA, with the average decomposition weight for each AT for AA and FCM, respectively. The proportion of normal pattern (AT1) was the greatest. Four patterns from AT2 to 5 exhibit superior hemifield defects, AT6 to 9 exhibit inferior hemifield defects, and AT10 exhibit double arcuate defects. AT11 represents temporal wedge defect, AT12 represents ring scotoma, and AT13 indicates total VF loss. AT14 to 16 are less likely to be associated with glaucomatous VF defects.

### 2. Comparison of performance in predicting MD changes between FCM and AA decomposition methods

We used 9,144 VF tests from 3,449 eyes of 3,449 patients as VF MD prediction models (Table 2). The mean ± SD of age, initial MD, and number of VF tests were 54.3 ± 16.8 years, −6.7 ± 7.8 dB, and 2.7 ± 1.3, respectively. The MSE values of the FCM decomposition were significantly lower than those of the AA decomposition (all *P* ≤ 0.001). The PCC values of FCM decomposition were significantly greater than those of AA decomposition (all *P* ≤ 0.001) (Table 4).

**Table 4:** Results from the machine learning models for predicting mean deviation change with the decomposition coefficient.

	AA	FCM		AA	FCM	
	*Mean squared error*		*P-value*	*Pearson correlation coefficient*		*P-value*
**K-Nearest Neighbor**	0.73 ± 0.04	0.33 ± 0.02	<0.001*	0.99 ± 0.00	1.00 ± 0.00	<0.001*
**Random Forest**	0.68 ± 0.04	0.24 ± 0.01	<0.001*	0.99 ± 0.00	1.00 ± 0.00	<0.001*
**Light Gradient Boosting**	0.25 ± 0.02	0.16 ± 0.01	<0.001*	1.00 ± 0.00	1.00 ± 0.00	<0.001*

*Indicates statistical significance

AA = archetypal analysis; FCM = fuzzy c-means

### 3. Comparison of diagnostic ability for detection of glaucomatous VF progression between FCM and AA decomposition methods

We compared the AUCs of slope_AA_ and slope_FCM_ in 229 eyes of 229 patients to detect VF progression. The most common diagnosis was primary open angle glaucoma (78.6%). The mean ± SD of age, initial MD, MD slope, VFI slope, and follow-up time were 54.7 ± 15.6 years, −5.4 ± 5.6 dB, −0.2 ± 0.9 dB/year, −0.7 ± 2.8%/year, and 31.1 ± 9.2 months, respectively (Table 3).

For the MD slope criterion, the AUCs of seven slope_FCM_ (43.8%) were significantly greater than those of slope_AA_ (all *P* ≤ 0.028). In addition, seven slope_FCM_ (AT1, AT4, AT5, AT10, AT12, AT13 and AT15) (43.8%) had AUCs greater than 0.7, whereas none of slope_AA_ did.

For the VFI slope criterion, the AUCs of three slope_FCM_ (17.6%) were significantly higher than those of slope_AA_ (all *P* ≤ 0.028). Three slope_FCM_ (AT5, AT10 and AT13) (17.6%) had AUCs greater than 0.7, whereas none of the slope_AA_ did.

For PLR criterion, the AUCs of six slope_FCM_ (37.5%) were significantly greater than those of slope_AA_. Only one slope_AA_ (AT1) (6.3%) had an AUC larger than 0.7, whereas six slope_FCM_ had AUCs larger than 0.7 (AT1, AT5, AT10, AT12, AT13, and AT15) (37.5%).

For all three VF progression criteria, the AUCs of three slope_FCM_ (AT5; superior altitudinal defect, AT10; double arcuate defect and AT13; total loss) were significantly greater than those of slope_AA_ (Table 5).

**Table 5:** The performance of the decomposition coefficient slope of each archetype determined by archetype analysis and fuzzy c-means for detecting glaucomatous visual field progression.

Archetype no.	Mean deviation slope					Visual Field Index slope					Pointwise linear regression				
	*AA*		*FCM*			*AA*		*FCM*			*AA*		*FCM*		
	*AUC*	*95% CI*	*AUC*	*95% CI*	*P-value*	*AUC*	*95% CI*	*AUC*	*95% CI*	*P-value*	*AUC*	*95% CI*	*AUC*	*95% CI*	*P-value*
**AT 1**	0.680	0.582–0.778	0.739	0.651–0.828	0.088	0.616	0.513–0.718	0.649	0.558–0.740	0.414	0.772	0.664–0.880	0.750	0.668–0.833	0.598
**AT 2**	0.546	0.418–0.675	0.649	0.527–0.772	0.088	0.590	0.455–0.724	0.531	0.398–0.664	0.650	0.530	0.365–0.695	0.608	0.453–0.764	0.238
**AT 3**	0.539	0.415–0.663	0.681	0.577–0.785	**0.028** ^*^	0.526	0.392–0.660	0.527	0.392–0.662	0.993	0.500	0.338–0.662	0.646	0.492–0.801	**0.047** ^*^
**AT 4**	0.531	0.411–0.651	0.737	0.646–0.829	**0.014** ^*^	0.564	0.442–0.687	0.590	0.465–0.715	0.792	0.535	0.399–0.670	0.697	0.566–0.829	0.078
**AT 5**	0.503	0.388–0.618	0.740	0.657–0.823	**<0.001** ^*^	0.554	0.426–0.683	0.722	0.623–0.821	**0.028** ^*^	0.581	0.435–0.728	0.800	0.717–0.882	**0.001** ^*^
**AT 6**	0.511	0.407–0.615	0.639	0.513–0.766	0.192	0.521	0.408–0.633	0.560	0.432–0.688	0.567	0.588	0.464–0.713	0.591	0.423–0.759	0.971
**AT 7**	0.510	0.390–0.631	0.622	0.493–0.751	0.101	0.563	0.435–0.691	0.532	0.396–0.667	0.607	0.642	0.508–0.775	0.592	0.429–0.756	0.491
**AT 8**	0.588	0.464–0.712	0.657	0.544–0.769	0.316	0.588	0.463–0.713	0.591	0.467–0.715	0.977	0.640	0.485–0.796	0.640	0.497–0.783	0.997
**AT 9**	0.582	0.436–0.701	0.600	0.466–0.734	0.845	0.537	0.409–0.665	0.521	0.393–0.649	0.872	0.579	0.426–0.733	0.511	0.355–0.667	0.971
**AT 10**	0.503	0.386–0.620	0.730	0.636–0.823	**0.001** ^*^	0.523	0.380–0.666	0.728	0.614–0.842	**0.004** ^*^	0.582	0.423–0.741	0.816	0.731–0.901	**0.008** ^*^
**AT 11**	0.686	0.582–0.790	0.560	0.426–0.693	0.038^*^	0.614	0.488–0.740	0.647	0.526–0.767	0.547	0.653	0.532–0.773	0.611	0.465–0.758	0.610
**AT 12**	0.599	0.492–0.707	0.776	0.695–0.857	**0.007** ^*^	0.609	0.483–0.736	0.704	0.592–0.816	0.341	0.572	0.422–0.722	0.825	0.742–0.908	**0.009** ^*^
**AT 13**	0.593	0.486–0.700	0.765	0.686–0.845	**0.001** ^*^	0.575	0.459–0.690	0.761	0.665–0.857	**0.006** ^*^	0.565	0.419–0.711	0.803	0.718–0.888	**0.003** ^*^
**AT 14**	0.516	0.398–0.634	0.651	0.527–0.775	0.092	0.563	0.450–0.676	0.578	0.446–0.710	0.844	0.573	0.439–0.707	0.657	0.497–0.817	0.363
**AT 15**	0.540	0.423–0.657	0.760	0.684–0.837	**0.001** ^*^	0.534	0.409–0.659	0.670	0.586–0.753	0.098	0.528	0.389–0.666	0.731	0.649–0.813	**0.005** ^*^
**AT 16**	0.593	0.483–0.702	0.592	0.473–0.710	0.987	0.610	0.495–0.712	0.600	0.491–0.710	0.884	0.614	0.466–0.762	0.522	0.375–0.669	0.501

*Indicates statistical significance

AA = Archetypal analysis; AT = archetype; AUC = area under the receiver operating characteristic curve; CI = confidence interval; FCM = fuzzy c-means

### 4. The relationship between AT decomposition coefficient slope and MD slope

**Fig 4:**
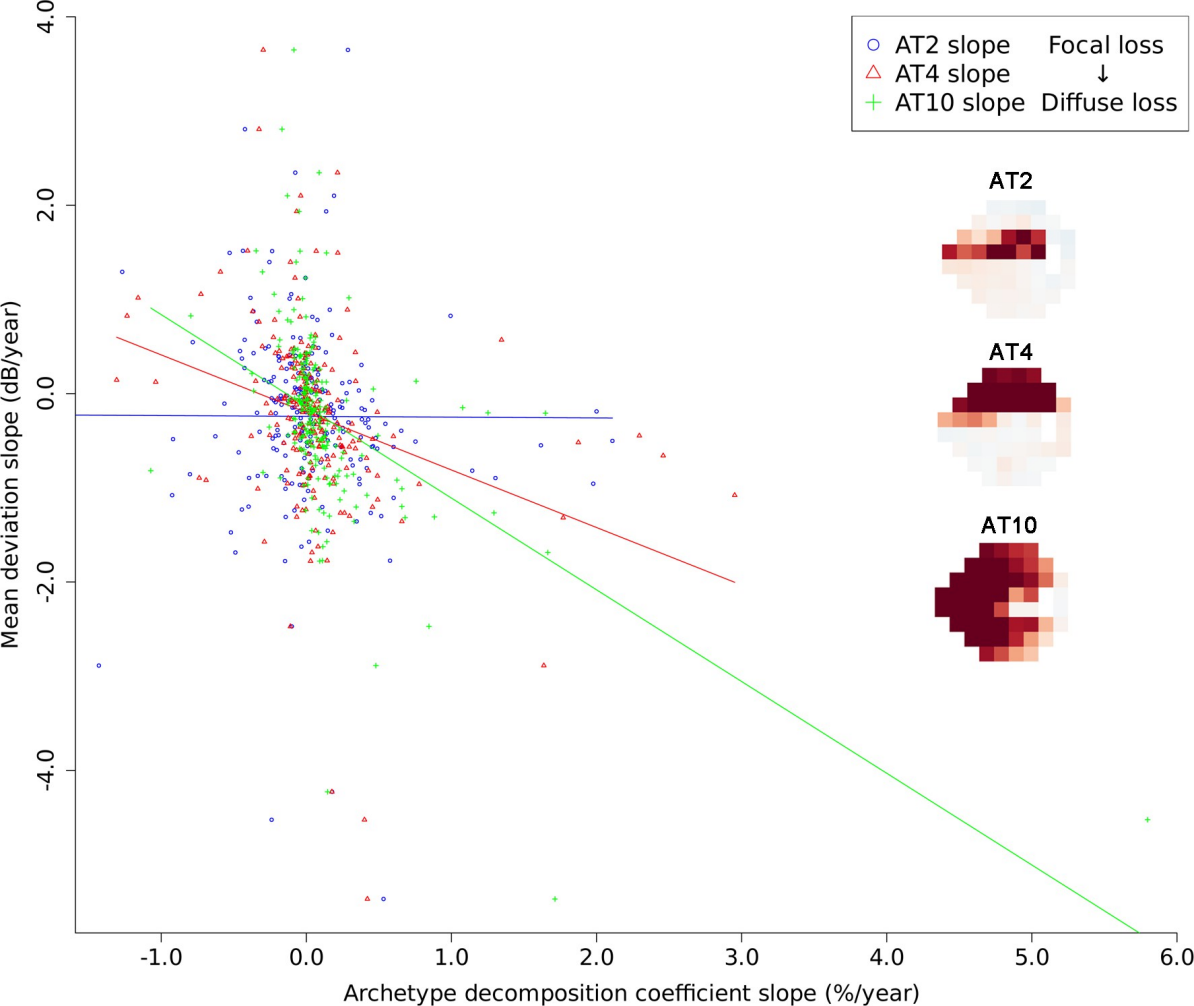
Scatter plots and fitted line for regression slope of the three representative archetypes (ATs) decomposition coefficient slope (slope_FCM_) against mean deviation (MD) slope for 229 eyes with more than five visual field (VF) tests. The magnitude of slope of the regression line for AT10 (Double arcuate defect), AT4 (central sparing superior hemifield loss), and AT 2 (superior arcuate defect) was 0.97, 0.61, and 0.01, respectively. The representative pattern of diffuse VF loss (AT10) exhibited a steeper slope compared to the less diffuse VF loss pattern (AT 4). The representative pattern of focal VF loss (AT 2) exhibited a nearly horizontal line.

Fig 4 illustrates scatter plots and fitted line for regression slope of the three representative ATs decomposition coefficient slope (slope_FCM_) against MD slope for 229 eyes with more than five VF tests. The three slope_FCM_ were selected based on their correlation coefficients with the MD slope. The correlation coefficient between slope_FCM_ of AT10 and MD slope was the highest (Spearman’s rho = −0.540, *P* < 0.001) among the three, while the correlation coefficient for slope_FCM_ of AT2 was the lowest (Spearman’s rho = −0.151, *P* = 0.023), and slope_FCM_ of AT4 represented an intermediate value (Spearman’s rho = −0.400, *P* < 0.001). The slope_FCM_ related to diffuse VF loss would be expected to appear as a steep diagonal line. In contrast, the slope_FCM_ associated with focal VF loss would be expected to appear close to a horizontal line, as it would have less effect on MD changes.

The representative pattern of diffuse VF loss (AT10, double arcuate defect), exhibited a steeper regression line (magnitude of slope = 0.97) compared to the slope of the less diffuse VF loss pattern (AT 4, central sparing superior hemifield loss) (magnitude of slope = 0.61). The representative pattern of focal VF loss (AT 2, superior arcuate defect) exhibited nearly horizontal line (magnitude of slope = 0.01).

### 5. Representative cases

**Fig 5:**
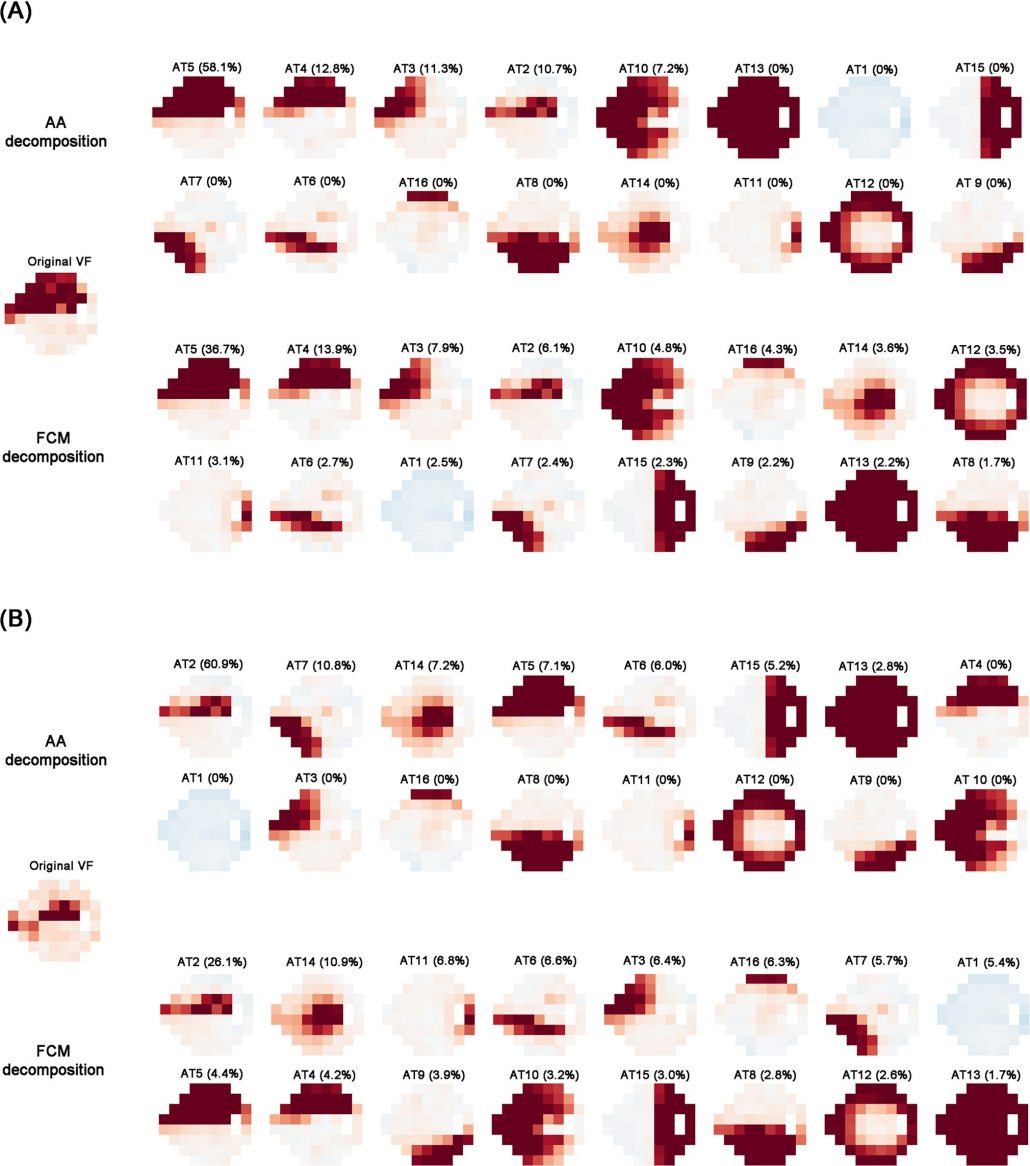
Two representative cases with visual field decomposition by archetypal analysis (left) and fuzzy c-means (right). (A) Case 1: The top five ATs and the order of decomposition were identical in both AA and FCM decomposition, even if their ratios were different. (B) Case 2: The order and ratio of ATs differed between AA and FCM decomposition except for the primary AT. Note that all visual fields were plotted in the right-eye format. AA = archetypal analysis; AT = archetype; FCM = fuzzy c-means.

Fig 5 depicts the quantitative decomposition of VF in two patients based on AA and FCM. The 16 ATs are arranged in order of high decomposition coefficient. The sum of the decomposition coefficients is 1 for both methods; however, the ratios of each pattern differed according to the AA and FCM methods.

Fig 5A illustrates a case in which the decomposed results obtained by AA and FCM were similar. AA and FCM has the same top five ATs while their decomposition coefficients differed. Fig 5B illustrates a case in which the orders of decomposition coefficient analyzed by AA and FCM were different while the most prominent ATs (AT2, superior arcuate defect) are identical. The inferonasal defect (AT7) is the second most frequent AT in AA, whereas central scotoma (AT14) is the secondary AT in FCM. Among the patterns obtained from the AA decomposition, the 8th through 16th most frequent ATs exhibit 0% ratio. In contrast, ATs obtained from FCM decomposition distribute some proportion across all 16 patterns.

## Discussion

In this study, we classified 16 characteristic patterns of 24–2 VF using AA, with 132,938 VFs from 32,553 eyes of 18,033 patients. We then decomposed 24–2 VF using FCM and compared its performance with that of AA decomposition. We found that FCM decomposition outperformed AA decomposition in predicting MD changes. In addition, FCM decomposition coefficient slopes demonstrated greater diagnostic ability in detecting VF progression than AA decomposition coefficient slopes. Lastly, we investigated the relationship between MD slope and FCM decomposition coefficient slopes to show whether VF loss of each AT are focal of diffuse.

In the longitudinal analysis, FCM provided a more informative decomposition than AA for predicting MD changes. Notably, slope_FCM_ outperformed slope_AA_ in detecting VF progression in majority of ATs. The number of ATs with AUCs larger than 0.7 was higher in slope_FCM_ than in slope_AA_. Most importantly, three slope_FCM_ (17.6%) demonstrated better diagnostic performance than slope_AA_ in all three different progression criteria; superior altitudinal defect, double arcuate defect, and total loss.

The results of this study are in agreements of those of our previous study (in preprint status: Yoo et al., Research square, August 08, 2022, doi:10.21203/rs.3.rs-1909859/v1). To overcome the inherent projection loss caused by converting high-dimensional 24–2 VF data into a convex combination of ATs, [11] we applied FCM decomposition in our previous study (in preprint status: Yoo et al., Research square, August 08, 2022, doi:10.21203/rs.3.rs-1909859/v1) using the original distance in the Euclidean space and we found that the hybrid approach combining AA and FCM provided more clinically relevant decomposition information than the conventional approach [32, 33]. In our previous study which decomposed 10–2 VF patterns of individual patient with FCM, FCM outperformed the AA-only approach in predicting MD changes. In predicting the 10–2 VF MD slope with baseline decomposition coefficient, the percentage of variance explained by FCM was higher than that explained by the AA-only approach (33.0% vs. 8.7%). The decomposition coefficients from FCM strongly improved the prediction of the 10–2 VF MD slope (Akaike and Bayes information criteria decrease by 17.62 and 12.96, respectively).

A steeper slope in the fitted regression between rate of change in decomposition coefficient slope (slope_FCM_) and MD (global VF loss) would indicate a more diffuse pattern of VF loss [34]. Double arcuate defect exhibited a steeper slope gradient than that of central sparing superior hemifield loss. The slope gradient of superior arcuate defect was close to zero indicating that this pattern is a focal defect which has little impact on global VF decay.

To include representative functional loss and present a clinically interpretable decomposition with a low reconstruction error, the number of ATs was set to 16 [35]. The 16 ATs may include non-glaucomatous VF patterns as well as glaucomatous VF patterns because patients visiting glaucoma clinics may have macular degeneration, secondary optic neuropathy, and cerebrovascular disease as well as glaucoma.

AT1 may be the normal VF, whereas AT2 to 13 are glaucomatous VF. AT14 to 16 may represent non-glaucomatous VF. AT 14 is central scotoma that may be associated with macular degeneration, optic neuropathy, and glaucoma [35–37]. AT15 is temporal hemianopsia that may be associated with brain diseases such as stroke or pituitary adenoma [4, 5, 38]. AT16 is superior depression that may be associated with ptosis [39]. Given that VF loss may result from a single disease or a combination of multiple diseases, a hybrid approach using AA and FCM decomposition methods may help clinicians discriminate glaucomatous VF loss from those related to different causes.

Our study has some limitations. First, while FCM decomposition does not assign a specific weight of 0% to any AT, AA decomposition gives more weight to ATs of interest and sets the weight of certain ATs to 0% during projection. In some cases, when representative VF patterns need to be identified rather than analyzing the overall VF patterns, AA decomposition may be more suitable. Second, we did not use other clinical data such as optic disc features, optical coherence tomography, and intraocular pressure for analysis. Third, our approach may not be suitable for detecting focal glaucomatous change such as single or a few visual field points progression within one visual field AT. In future studies, we will improve our hybrid model by adding clinical data.

In summary, a hybrid approach for analyzing 24–2 VF loss using AA and FCM visualizes 24–2 VF loss in characteristic patterns, improving the understanding of individual patients’ 24–2 VF tests through lossless decomposition. Additionally, demonstrating the progression of each AT in a longitudinal assessment can provide clinicians with useful information regarding the progression rate according to each VF defect pattern. We believe the hybrid approach of AA and FCM used in this study represents a pioneering method in ophthalmology research, and it is expected to be helpful as an initial investigation for future studies.
